# Establishment and Characterization of a Tumor Stem Cell-Based Glioblastoma Invasion Model

**DOI:** 10.1371/journal.pone.0159746

**Published:** 2016-07-25

**Authors:** Stine Skov Jensen, Morten Meyer, Stine Asferg Petterson, Bo Halle, Ann Mari Rosager, Charlotte Aaberg-Jessen, Mads Thomassen, Mark Burton, Torben A. Kruse, Bjarne Winther Kristensen

**Affiliations:** 1 Department of Pathology, Odense University Hospital, Denmark, Odense C, Denmark; 2 Department of Clinical Research, University of Southern Denmark, Odense C, Denmark; 3 Department of Neurobiology Research, Institute of Molecular Medicine, University of Southern Denmark, Odense C, Denmark; 4 Department of Neurosurgery, Odense University Hospital, Odense C, Denmark; 5 Department of Clinical Genetics, Odense University Hospital, Odense C, Denmark; Swedish Neuroscience Institute, UNITED STATES

## Abstract

**Aims:**

Glioblastoma is the most frequent and malignant brain tumor. Recurrence is inevitable and most likely connected to tumor invasion and presence of therapy resistant stem-like tumor cells. The aim was therefore to establish and characterize a three-dimensional in vivo-like in vitro model taking invasion and tumor stemness into account.

**Methods:**

Glioblastoma stem cell-like containing spheroid (GSS) cultures derived from three different patients were established and characterized. The spheroids were implanted in vitro into rat brain slice cultures grown in stem cell medium and in vivo into brains of immuno-compromised mice. Invasion was followed in the slice cultures by confocal time-lapse microscopy. Using immunohistochemistry, we compared tumor cell invasion as well as expression of proliferation and stem cell markers between the models.

**Results:**

We observed a pronounced invasion into brain slice cultures both by confocal time-lapse microscopy and immunohistochemistry. This invasion closely resembled the invasion in vivo. The Ki-67 proliferation indexes in spheroids implanted into brain slices were lower than in free-floating spheroids. The expression of stem cell markers varied between free-floating spheroids, spheroids implanted into brain slices and tumors in vivo.

**Conclusion:**

The established invasion model kept in stem cell medium closely mimics tumor cell invasion into the brain in vivo preserving also to some extent the expression of stem cell markers. The model is feasible and robust and we suggest the model as an in vivo-like model with a great potential in glioma studies and drug discovery.

## Introduction

Glioblastomas are the most malignant brain tumors with inevitable tumor recurrence after treatment. Two of the crucial factors believed to be responsible for tumor recurrence are the invasive properties of these tumors [1] combined with the treatment resistant tumor stem-like cells [2–4].

Glioblastomas have pronounced invasive properties [1] with tumor cells often spreading into corpus callosum and reaching the contralateral hemisphere or other distant brain regions [5]. The invasive cells escape surgery and changes in the invasive cells influencing apoptosis and proliferation may explain why they survive chemotherapy [6]. Similar mechanisms might explain therapeutic resistance of tumor stem-like cells towards both chemotherapy [7] and radiation [2, 8]. Interestingly, studies [9, 10] have suggested the invasive cells to have stem-cell properties, being more aggressive than their non-invasive counterpart [11]. In vivo-like in vitro models for investigation of invasion integrating the role of tumor stem-like cells into the models are therefore very import, but may have several limitations. In some studies migration on the bottom of plastic plates with different coatings have been used [12, 13] as well as Boyden chamber assays monitoring cell invasion through a membrane [14, 15]. A more in vivo-like model would be to introduce the tumor cells into cultured brain tissue. Hereby, the structure of the brain is preserved and a more optimal microenvironment for studies of tumor cell invasion is created.

The aim of the present study was therefore to establish and evaluate a tumor stem cell-based in vitro invasion model by implanting glioblastoma stem cell-like containing spheroids (GSS) into rat organotypic brain slices cultured in stem cell promoting medium. This model comprises the use of the spheroid model used in glioma stem-cell research [16–19] and the brain slice culture model used extensively in neuroscience [20–23]. In order to evaluate the model, invasion in the in vitro model was compared with invasion in the in vivo situation, xenografting tumor cells from GSS into the brain of immunodeficient mice. Moreover, an immunohistochemical comparison of in vitro cultured free-floating GSS, in vivo implanted GSS and GSS implanted in vitro was performed, hypothesizing a possible therapeutically relevant phenotypic shift related to proliferative potential and tumor stem cell properties.

## Material and Methods

### Tumor cell lines and primary tumor tissue

Glioblastoma tissue was collected and processed by manual dissociation into small tissue fragments. These fragments were cultured until they rounded up to form spheroids, where after the spheroids were trypsinated and allowed to form new spheroids. Three glioblastoma GSS cultures (T78, T86 and T87) were established in our laboratory [17] and used in the present study (T78 is referred to as GBM5 and T86 as GBM9 in [17]). Besides the GSS cultures the commercial glioblastoma cell line U87 (from European Collection of Cell Cultures (ECACC)) was used. All cell lines were cultured in serum-free medium composed of Neurobasal A (Invitrogen), 2% B27 supplement without vitamin A (Invitrogen), N2 (Invitrogen), 1% glutamine (Cambrex), 20 ng/mL EGF (Sigma-Aldrich), 20 ng/mL bFGF (Trichem A/S), and 1% penicillin-streptomycin (Cambrex). The cells were cultured at 36°C in a standard tissue culture incubator (95% humidity, 95% air, and 5% CO2). In order to determine the growth rates, cells were seeded in a 6-well plate (45000 cells/well) in 2 ml serum-free medium. The cell numbers were estimated in triplicates at day 1–5. The three GSS cultures were further characterized by a spheroid formation assay at clonal density [24, 25], karyotyping, a differentiation assay [24, 25], mIDH1 immunohistochemistry [26, 27] and MGMT analyses (QIAamp®DNA FFPE Tissue kit, the EpiTect Plus DNA Bisulfite Kit and MGMT Pyro kit, all Qiagen) as well as by in vivo xenografting of the cells into immunodeficient mice. Molecular subtypes were determined by single sample prediction using nearest centroid method reported by Verhaak et al. [28]. For T87 this resulted in a very clear proneural subtype (CC = 0.48) and for T86 a classical subtype (CC = 0.12), whereas T78 was of mesenchymal subtype (CC = 0.11). Part of the characterization comprising the capability of spheroid formation in vitro at clonal density and tumor formation in vivo has previously been published for T78 and T86 [17].

### Organotypic brain slice cultures and co-cultures

Organotypic corticostriatal slice cultures were prepared by the interface method [23, 25, 29] and grown for the first three days in a serum-based culture medium as earlier described [23, 29]. Thereafter the medium was changed to serum-free medium as used for culturing the GSS cultures.

Spheroids (200–400 μm) were incubated in 25 μg/ml DiI solution (DiI, Molecular Probes, Invitrogen) for 24 h, washed, and implanted into the brain slice cultures in the area between cortex and striatum close to corpus callosum. To enable capture and correct placement onto the brain slice cultures only spheroids of 200–400 μm were used. The tumor cells were visualized using confocal microscopy (Nikon, Inverted Microscope, ECLIPSE TE2000-E with time-lapse function and perfect focus system). After 1 h of incubation confocal z-stacks with 20 μm steps were recorded, before superimposing the z-stacks to one image representing the entire spheroid. This procedure was repeated at day 3 and day 6, where after the co-cultures were fixed in 10% formalin and paraffin embedded.

Additionally, time-lapse experiments were performed following the in vitro invasion of tumor cells into the brain slice cultures using DiO labeled T78 and T86 spheroids (DiO, Molecular probes, Invitrogen). A z-stack was recorded every half hour for 11 hours and 30 minutes and hereafter every hour for 48 hours. This resulted in time-lapse movies showing the tumor cell invasion into the brain tissue in the first approximately 60 hours after implantation. The co-cultures were cultured in a CO_2_ Microscope Cage Incubator (Okolab, Italy) (36°C, 95% humidified air, and 5% CO_2_) mounted on the confocal microscope.

### Xenograft model

Female Balb c nu/nu mice 7–8 weeks of age were anesthetized and tumor cells injected into the brain as earlier described [17]. Two survival protocols were used to monitor tumor growth. In the first protocol (max survival), the mice were euthanized upon symptoms and the brains investigated. In the second protocol (short survival), we aimed to investigate an earlier stage of tumor growth and mice were euthanized 30 days after implantation. The brains were removed immediately after death and fixed in 10% formalin for 24 h. Before paraffin embedding the brains were divided by a coronal section at the injection site in an anterior and posterior part. Histological sections of the resulting paraffin blocks included two coronal sections of the brain.

### Measurements in confocal images

After superimposing the confocal z-stacks, the area of the spheroids was measured using the software Visiomorph (Visiopharm, Hørsholm, Denmark). The spheroids were outlined at the spheroid boarder identifying the beginning of the invasion zone. The area of the invasive cells was also measured in Visiomorph using a classifier identifying the area of DiI staining representing only the invasive cells and not the spheroids. The invasion distance in the confocal images was not measurable because of the small field of view.

### Measurements in histological sections

The tissue sections stained with vimentin and CD56 were scanned using the whole slide scanner (NanoZoomer 2.0-HT slide scanner, Hamamatsu). The area of the spheroids in vitro and the tumors in vivo were measured using the program NanoZoomer Digital Pathology Version 2.3.11 from Hamamatsu. Using an area tool the spheroid or bulk tumor area without invasion were outlined and measured. The invasion area was measured as being the tumor cell area found outside the spheroids or tumor bulk using the software Visiomorph by making a classifier identifying the area of positive vimentin and CD56 staining subtracting the area of the spheroid or tumor bulk. The longest invasion distance was found using a linear measurement tool, measuring the perpendicular distance from the border of the spheroid or tumor bulk to the invasive front of the tumors.

### Immunohistochemistry

Immunohistochemical analysis was carried out on the paraffin-embedded spheroids, mice brains from in vivo xenografts and co-cultures as described previously [23, 30, 31] using the antibodies vimentin, CD56, CD133, nestin, podoplanin and Ki-67. The Ki-67 labeling index (Ki-67 LI) was determined using the software program Tissuemorph (Visiopharm, Hørsholm, Denmark). The stem cell markers were assessed by semi-quantitative scoring from 0–3, with 0 being negative staining, 1 weak staining, 2 moderate staining and 3 extensive staining. The staining of the invasive cells was not scored but it was investigated whether positive cells were found outside the spheroids.

### Ethics

The official Danish ethical review board named the Regional Scientific Ethical Committee of the Region of Southern Demark approved the use of human glioma tissue (permission J. No. S-VF-20040102) in the current study. Written informed consent was obtained from all participants.

The use of animals for organotypic brain slice cultures was approved by The Animal Experiments Inspectorate in Denmark (permission J. No. 2008/561-1572). The rats (newborn wistar rats, Taconic Denmark, n = 53) were decapitated and the brains were removed.

The use of animals for glioblastoma mice (Female Balb c nu/nu mice 7–8 weeks, Taconic Denmark, n = 21) xenografts was approved by The Animal Experiments Inspectorate in Denmark (permission J. No. 2008/561-1572 and J. Nr. 2013-15-2934-00973). Mice were anesthetized by a subcutaneous injection with a mixture of hypnorm (fentanyl, 0,315 mg/ml; fluanisone, 10 mg/ml) and midazolam (0.12 ml/10g). The mice were euthanized in a carbon dioxide chamber upon symptoms such as weight loss (20% loss of body weight) or general poor state including lethargy, hunched posture and failure to groom.

The animals were housed according to national guideline (National declaration for animal experiments 2013), and had free access to food and water.

### Statistics

Data were analyzed using one-way ANOVA with Bonferroni post test to compare all data sets. Statistical significance was defined as *P<0.05, **P<0.01, ***P<0.001. The survival of the mice in the in vivo xenograft model was analyzed using a Kaplan-Meier plot. All statistics were carried out using Graph Pad Prism 5.0 (Graphpad Software, San Diego, California USA).

## Results

### In vitro characterization of the GSS cultures

**Fig 1:**
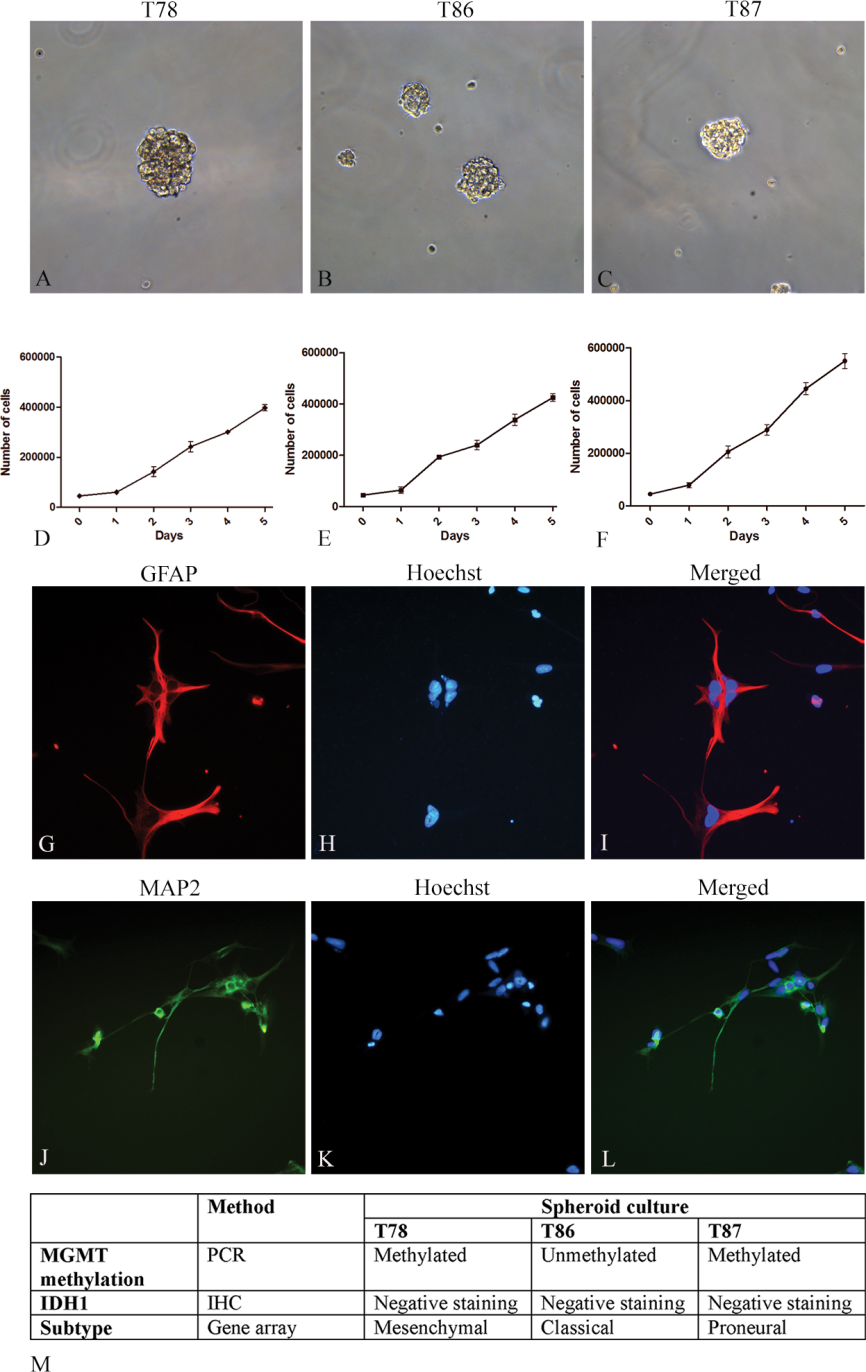
Characterization of GSS cultures. The three GSS cultures were cultured in serum-free medium as spheroids, which upon trypsination to single cells developed new spheroids (A-C). Cells were seeded and the cell number estimated at day 1–5 (n = 3) (D-F). Differentiation assays were performed showing expression of the astrocytic marker GFAP (G-I) and the neuronal marker MAP2 (J-L), here illustrated for T78. The MGMT status for each culture was determined by PCR and T78 and T87 were found to be methylated whereas T86 was unmethylated (M). All three GSS cultures were derived from IDH1-negative tumors representing primary glioblastomas (M). Scalebar 100 μm (A-C) and 50 μm (J-L).

All three GSS cultures formed tumor spheroids at passage numbers 5 to 12 used in the present study (Fig 1A–1C). The tumor cells were all derived from IDH1-negative tumors suggesting that they were primary glioblastomas (Fig 1M) [32]. Upon karyotyping the three cell lines all showed gain of chromosome 7 and in T86 and T87 loss of chromosome 10, characteristics commonly observed in glioblastomas. The cell growth of T87 and T86 was similar, whereas T87 had a significant higher growth rate (Fig 1D–1F). For comparison of growth rates of all GSS cultures and U87 see supplementary data (S1 Fig). Hypermethylated CpG islands were found in the MGMT promoter region of U87, T78 and T87 but not for T86 (Fig 1M). The differentiation assay (data shown for T78) showed differentiation into the astrocytic and neuronal lineage as identified by GFAP (Fig 1G–1I) and MAP2 (Fig 1J–1L), positive cells, respectively.

### Confocal imaging of migrating tumor cells

**Fig 2:**
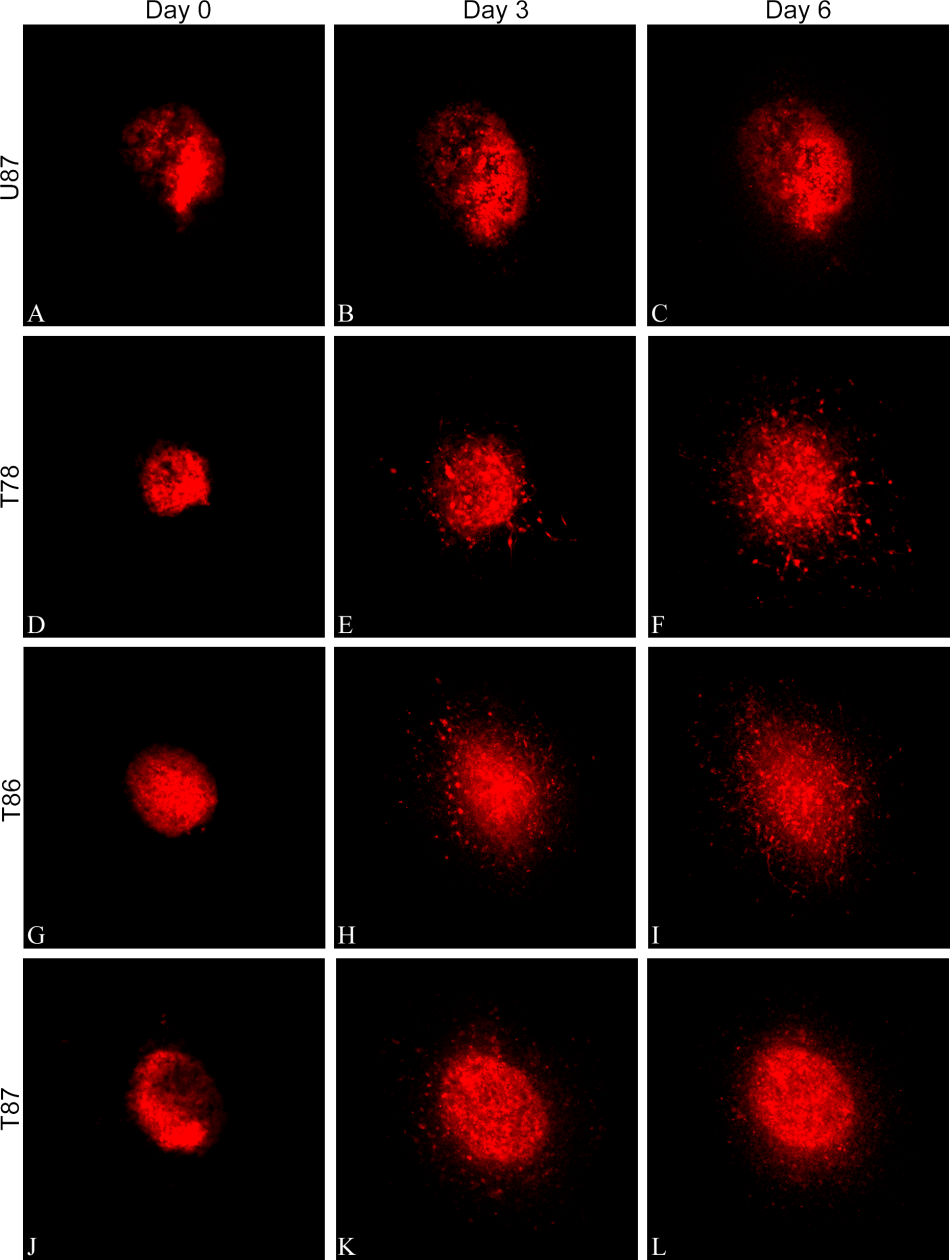
Confocal images of spheroid invasion. Confocal z-stacks were recorded on day 0, one hour after implantation of DiI-labeled spheroids into the brain slice cultures and after 3 and 6 days. The z-stacks were superimposed into one image representing the entire spheroid. The glioma cell lines U87 showed no particular invasion of cells into the brain slice cultures (A-C), whereas the three GSS cultures all showed pronounced invasion of tumor cells into the brain tissue (D-L). Scalebar 100 μm.

**Fig 3:**
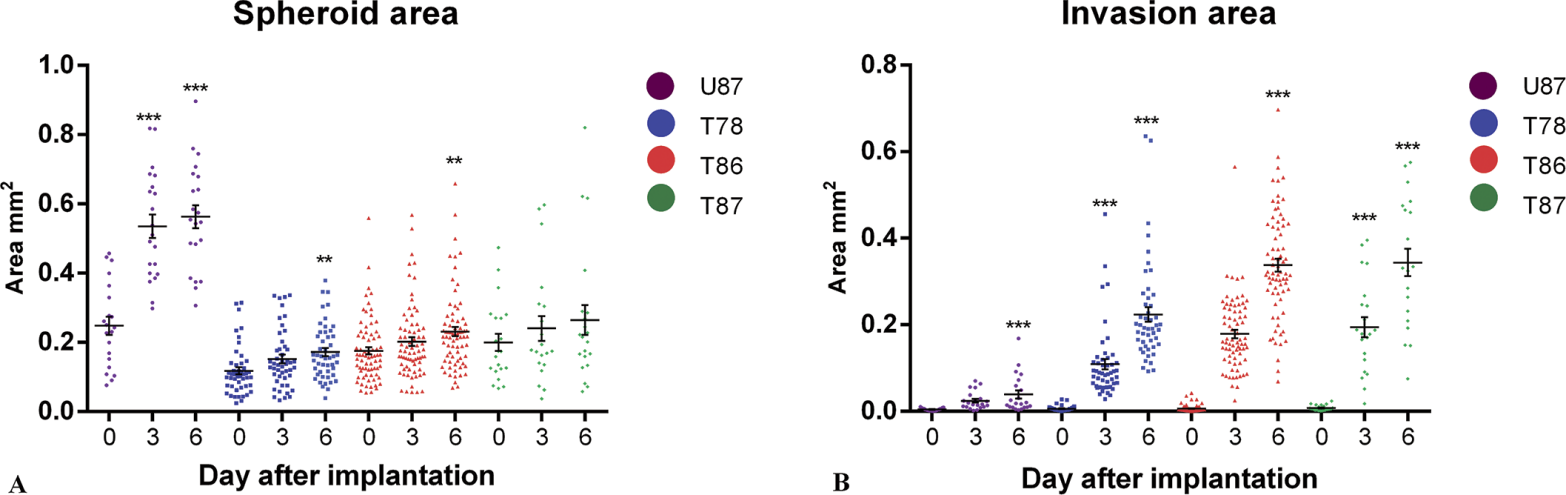
Quantitation of area and invasion in confocal images. The spheroid area was measured in the confocal images on day 0, 3 and 6 (T78: n = 48, T86: n = 73, T87: n = 21, U87: n = 21) (A) as well as the invasion area outside the spheroid (B). Data are shown as means ± SEM, and statistical significance *P<0.05, **P<0.01, ***P<0.001 was investigated using ANOVA with Bonferroni correction for comparison with day 0.

Confocal images of the DiI-labeled spheroids implanted into brain slice cultures were recorded and supposed invasion was seen for all three GSS cultures and for the U87 cell line (Fig 2A–2L). The area of U87 increased significantly from day 0 to day 3 and 6 (Figs 2A–2C and 3A), whereas only a slight increase in the area of the GSS cultures T78 (Figs 2D–2F and 3A), T86 (Figs 2G–2I and 3A) and T87 (Figs 2J–2L and 3A) was significant for T78 and T86 on day 6. A small but significant increase in area of the supposedly invasive cells was seen for U87 after 6 days (Fig 3B). For T78, T86 and T87 (Fig 3B), there was a pronounced increase being significant after both 3 and 6 days (Fig 3B).

Time-lapse movies showed clearly how cells migrated into the brain tissue, but cellular reorganization in the center of the spheroids was also revealed. Furthermore, what appeared as cell divisions were seen for T86 (S2 Fig).

### Immunohistochemical evaluation of expression of glial and neuronal markers in brain slice cultures

In both the cortex and striatum of brain slice cultures, cells with astroglial and neuronal morphologies expressed the astrocytic marker GFAP and the neuronal markers MAP2 and NeuN, respectively suggesting a normal cell composition of both brain regions (S3 Fig).

### Immunohistochemical detection of tumor cell invasion in vitro

**Fig 4:**
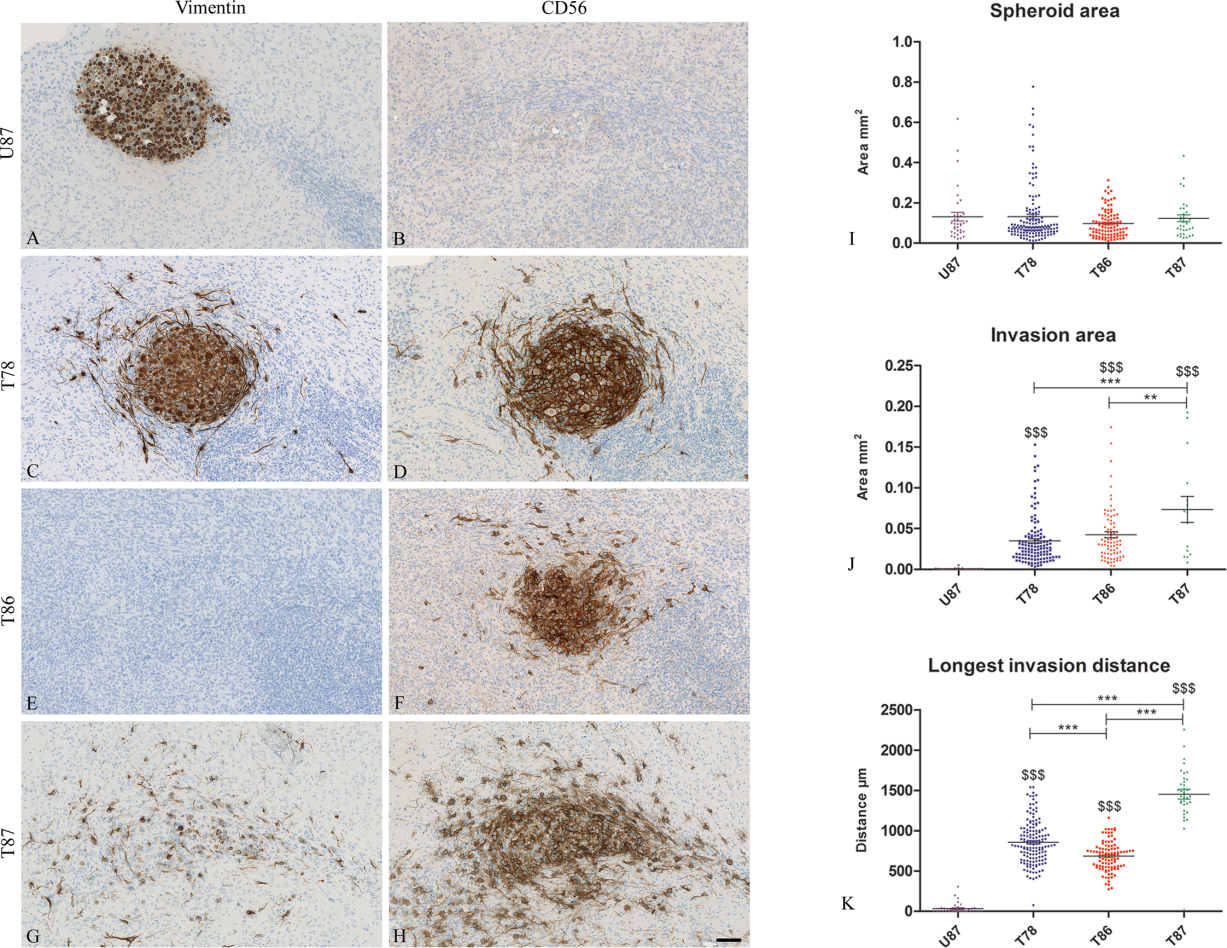
Invasion in the in vitro invasion model. Thin 3 μm sections of spheroids implanted into brain slice cultures were stained by immunostained using anti-human vimentin (A, C, E, G) and CD56 (B, D, F, H) antibodies. Co-cultures with both U87 spheroids (A, B), T78 (C, D), T86 (E, F) and T87 spheroids (G, H) were stained. Spheroid area (I), invasion area (J) and longest invasion distance (K) were measured on the immunostained sections (T78: n = 147, T86: n = 98, T87: n = 35, U87: n = 36). Data are shown as means ± SEM, and statistical significance */$ P<0.05, **/$ $ P<0.01, ***/$ $ $ P<0.001 was investigated using ANOVA with Bonferroni correction for comparison of all groups. $ is comparison of the GSS cultures to U87. Scalebar 100 μm (A-H).

After culturing, three μm sections of co-cultures were immunohistochemically stained with anti-human vimentin and CD56 (Fig 4). The borders of the U87 spheroids were well defined and no invasion was seen in the brain slice cultures. The U87 spheroids stained positive for vimentin (Fig 4A) but was negative for CD56 (Fig 4B). In contrast an invasive tumor cell phenotype with elongated cell morphology and membrane protrusions was derived from all GSS cultures (Fig 4C, 4D and 4F–4H). Histology revealed that spheroids implanted into brain slice cultures appeared viable after implantation as seen by the nuclear counter staining (Fig 4 and S4–S7 Figs) and both central and peripheral aspects of the spheroids displayed proliferative activity (S4 Fig).

The T78 spheroids stained positive for both vimentin (Fig 4C) and CD56 (Fig 4D). The T86 spheroids stained positive for CD56 (Fig 4F) but were negative for vimentin (Fig 4E). The implantation of T87 into the brain slice cultures was difficult, since some of the spheroids tended to fragment upon implantation into the brain slice cultures. These spheroids were therefore placed on top of the slice cultures instead of being embedded into the tissue. This may explain the less well defined margin of these spheroids (Fig 4H) in contrast to what was seen for T78 and T86 (Fig 4D and 4F). The invasion of T87 into the brain tissue, however, seemed to be more pronounced. T87 stained positive for both vimentin (Fig 4G) and CD56 (Fig 4H) but the vimentin staining was not found in all tumor cells compared to CD56.

No significant differences were found between the spheroid areas of the different cultures (Fig 4I) confirming that the intention of implanting spheroids of similar size was obtained. The invasion area of T87 was significant larger than the areas for T78, T86 and U87 (Fig 4J). The longest invasion distance was obtained for T87 as well (Fig 4K).

### Tumor development and invasion in mice

**Fig 5:**
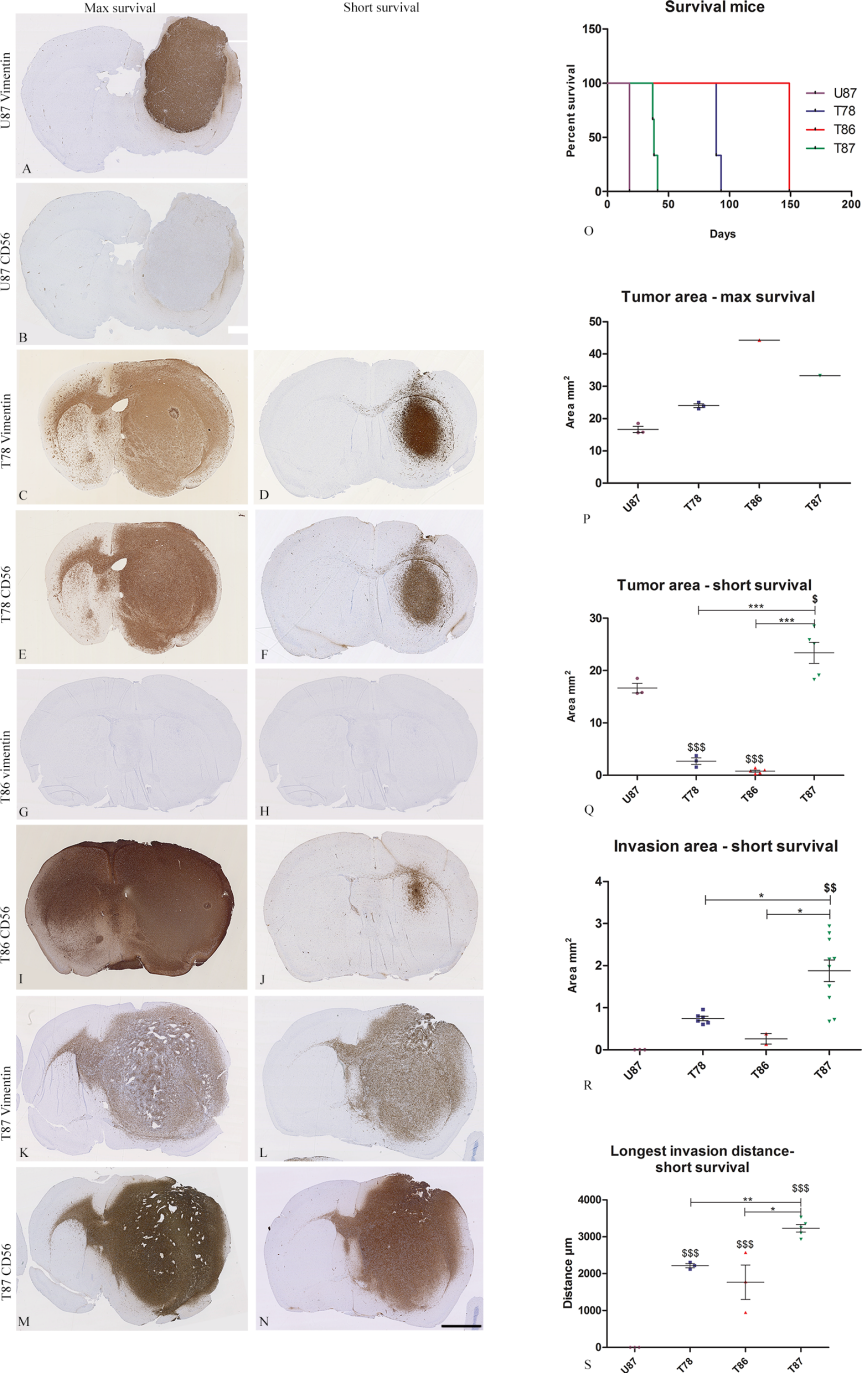
Tumor development in the in vivo xenografts. Two in vivo xenograft protocols were used for implantation of U87 and the GSS cultures in nude mice. A “max survival” (A and B (n = 3), C and E (n = 3), G and I (n = 1), K and M (n = 3)) and a “short survival” (D and F (n = 3), H and J (n = 3), L and N (n = 7)) protocol. Histological sections from the mice brains were immunostained with anti-human vimentin and CD56 for identification of the tumor cells. The survival (O) was recorded and the following measurements were performed: tumor area (max survival (P) and short survival (Q)), invasion area (short survival (R)) and longest invasion distance (short survival (R)). Data are shown as means ± SEM, and statistical significance */$ P<0.05, **/$ $ P<0.01, ***/$ $ $ P<0.001 was investigated using ANOVA with Bonferroni correction for comparison of all groups. $ is comparison of the GSS cultures to U87. Scalebar 2 mm (A-N).

Two protocols of implantation were used. In the first protocol (max survival) the mice were implanted with U87 and the three GSS cultures and allowed to live until symptoms appeared. To observe the tumors at an earlier stage of development, a second protocol (short survival) was used. Only GSS cultures were implanted since the animals were euthanized on day 30 after implantation (Fig 5).

With the first protocol, mice implanted with U87 cells were the first to have symptoms and were euthanized already after 19 days. Mice implanted with the GSS culture T87 had symptoms after 41 days. Two of the three mice implanted with T87 died before being euthanized resulting in a tissue quality not suitable for immunohistochemical staining. Mice implanted with T78 were euthanized after 89 days, whereas the mouse with T86 was euthanized after 149 days. Two of the three mice implanted with T86 died after surgery (Fig 5O). Thus survival curves should be used with caution due to the low number of mice included.

All tumors were stained with anti-human vimentin and anti-human CD56 antibodies and the expression pattern of the antibodies for each cell culture were identical to the pattern found in the in vitro model (Figs 4A–4H and 5A–5N), although the immunohistochemical stainings is only indicative due to the low number of mice. U87 grew as a solid tumors comprising the majority of one hemisphere without any invasive characteristics (Fig 5A), whereas the three GSS cultures in the max survival protocol all formed large infiltrative tumors migrating through the corpus callosum to the contralateral hemisphere (Fig 5C, 5E, 5I, 5K and 5M). In the max survival protocol T86 formed the largest tumor followed by T87, T78 and U87 (Fig 5P). In the short survival protocol T87 formed tumors approximately having the size of the tumors developed in the max survival protocol but being significantly larger than the tumors developed upon implantation with T78 and T86 (Fig 5Q). U87 was significantly smaller than T87 but larger than both T78 and T86 (Fig 5Q). The same U87 data are shown on both graphs (Fig 5P and 5Q) since mice implanted with U87 cells had a short max survival.

The invasion area and the distance to the invasive margin were measured in the tumors developed using the short survival protocol and for U87 tumors using the max survival protocol. The invasion area of T87 was significant larger than the invasion area of T78, T86 and U87 (Fig 5R). Likewise, the distance of invasion was significantly longer for T87 compared to T78, T86 and U87 (Fig 5S).

### Comparison of proliferation and stem cell marker expression

**Fig 6:**
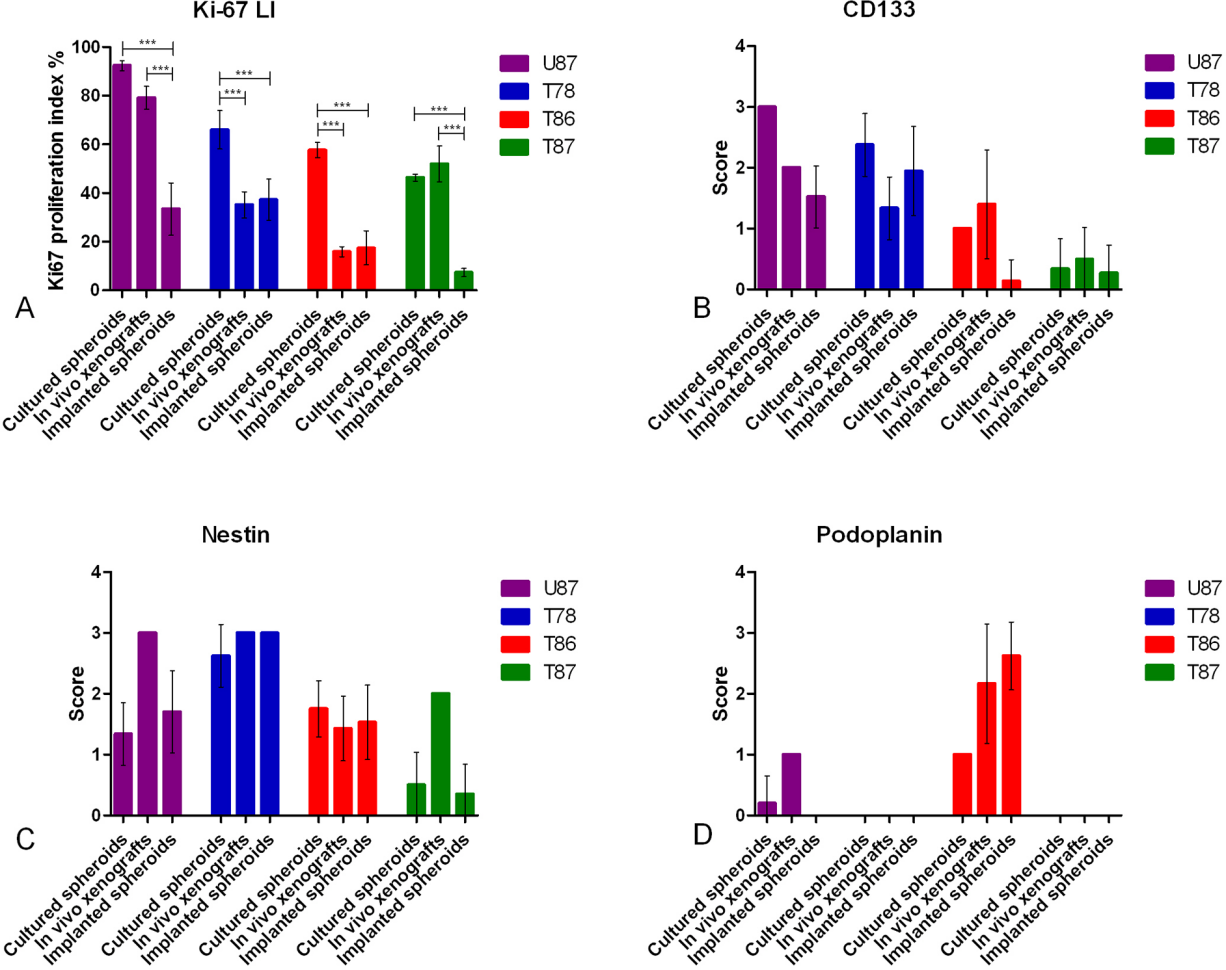
Ki-67 labeling index and scoring of stem cell markers in cultured spheroids, in vivo xenografts and implanted spheroids. Ki-67 LI (A) was measured in cultured spheroids, in vivo xenografts and implanted spheroids using ki-67 stained histological sections. A semi-quantitative scoring was performed for CD133 (B) nestin (C) and podoplanin (D). Data are shown as means ± SEM, n = 4–9 for cultured spheroids, n = 2–12 for in vivo xenografts, n = 5–58 for implanted spheroids, and statistical significance *P<0.05, **P<0.01, ***P<0.001 was investigated using ANOVA with Bonferroni correction for comparison of all groups.

Ki-67 positive nuclei were found in all spheroids, at the central tumor site in all xenografts and in all implanted spheroids (Fig 6A and S4 Fig). Moreover, Ki-67 immunohistochemical staining labeled invasive cells in vitro (S4 Fig) and in vivo (data not shown) for all three GSS cultures. Having the vimentin and CD56 tumor cell stainings as a reference, fewer of the invasive compared to the central tumor cells expressed Ki-67. For U87 a very high Ki-67 LI was found in both the cultured spheroids (92%) and the tumors in vivo (79%), whereas a lower Ki-67 expression was found in the spheroids implanted into the brain slice cultures (33%) (Fig 6A). Interestingly, both T78 and T86 had a high Ki-67 LI in the cultured spheroids (66% and 58%), whereas a decreased and similar proliferation index were found in the tumors in vivo (35% and 16%) and in the in vitro implanted spheroids (37% and 17%) (Fig 6A). For T87 a different proliferation pattern was seen with the highest Ki-67 LI in the cultured spheroids (46%) and in the tumors in vivo (52%) compared to the Ki-67 LI in the implanted spheroids (7%) (Fig 6A).

The stem cell markers CD133, nestin and podoplanin were analyzed in the three models showing different staining patterns both in between the models but also between the GSS cultures used. Due to some co-reactivity of the CD133 and nestin antibodies in mouse and rat tissue a separate evaluation of the protein expression in invasive cells was not performed.

In U87 and T78 a trend towards a higher expression of CD133 was found in the cultured spheroids, whereas for both T86 and T87 a trend towards a higher CD133 expression in the tumors in vivo was found (Fig 6B and S5 Fig).

Regarding nestin, a trend towards the highest expression in U87 and T87 was found in the tumors in vivo, whereas both T78 and T86 showed a similar expression in all three models (Fig 6C and S6 Fig). The nestin expression in the core spheroid and the invasive cells in vitro was very pronounced for T78, appearing as if most of the tumor cells expressed nestin. A less prominent nestin staining of T86 and T87 invasive cells was found (S6 Fig).

Regarding podoplanin, U87 tumors in vivo displayed only a few positive cells and for both T78 and T87 no podoplanin expression was found in tumor cells in any of the three models. T86 showed intense staining in both the tumors in mice and in the implanted spheroids (Fig 6D and S7 Fig). The positive staining identified in T78 and T86 tumors in vivo also comprised the blood vessels (S7 Fig). In the T86 cultures a few invasive cells stained positive both in vivo and in vitro, however, the majority of invading cells did not express podoplanin (S7 Fig).

## Discussion

In the present study an in vivo-like in vitro invasion model was established using stem cell medium. The model was based on GSS cultures implanted into rat brain slice cultures. Invasion in this model was for the first time compared with invasion in vivo and important similarities were found. However, when comparing the stem cell-like phenotype of the tumor cells in the three different models, the phenotype was found to vary both between models and GSS cultures. These results suggest that the established in vitro invasion model is a valuable tool in invasion studies but phenotypic differences in stem cell features may be present between the models.

### In vitro invasion

The in vitro invasion model established in the present study showed invasion patterns comparable with the invasion of tumor cells implanted in vivo suggesting this model to be in vivo-like. The earlier described circumscribed growth of U87 in vivo [16, 33, 34] in contrast to the infiltrative growth of the GSS cultures was also demonstrated in the in vitro invasion model established in the present study adding profound evidence that the model is a valid system to use for invasion studies. In more traditional in vitro assays like the wound healing assay and the transwell migration assay, U87 has been described as a migrating cell line [35–37], thereby suggesting that our model may have an advantage in terms of better mimicking the mechanisms playing a role in vivo. The explanation is most likely due to invasion being a process with mutual interaction between tumor cells and cells in the brain slices (neurons, astrocytes and microglial cells) and this is well mimicked in the spheroid-brain slice assay as opposed to many other assays. Organotypic brain slice cultures implanted with glioma cells have been used before [23, 32, 38–43] but the present study is the first to use GSS cultures and stem cell-like growth conditions incorporating the important stem cell aspect in the model. The stem cell-like growth condition seemed suitable for brain slice cultures since the main glial and neuronal compartments were preserved like previously shown in brain slice cultures cultured in conventional serum-free medium with low growth factor concentrations [44, 45]. Furthermore, the present study is one of the first to use both confocal imaging and subsequent immunohistochemistry confirming convincingly in 3 μm thin histological sections, the findings observed in the confocal images by quantitative histological methods. Previously, tissue fragments from biopsy tissue have been implanted into brain slice cultures [39, 43], providing in principle an even more in vivo-like model with the tumor tissue never being cultured before implantation into slice cultures. The same is the case when implanting primary organotypic spheroids as recently reported by our group [23]. The drawback of these approaches is the heterogeneity of the primary tissue and the limited possibility of repeating the experiments. In contrast using our approach, GSS spheroids are less heterogenic and can be propagated to large numbers and used for repeated experimental setups. Moreover neither the model based on tissue fragments [39, 43] nor the model based on primary spheroids [23] were established using stem cell medium but instead conventional serum containing medium.

### Proliferation and invasion

The tumor cell proliferation measured by the Ki-67 LI seemed to be highly affected by the culturing conditions. For both U87 and the three GSS cultures, the Ki-67 LI was significantly higher in the cultured spheroids than in the spheroids implanted in vitro into the rat brain slice culture. T78 and T86 were the cultures most easily implanted into the brain slices and interestingly, the in vivo Ki-67 LI level of these two GSS cultures was also found in the in vitro implanted spheroids. A decrease in proliferation was expected both in the tumors in vivo and in the in vitro invasion model compared to free-floating spheroids, since the spheroids were taken from a culturing condition supporting spheroid formation to a substrate supporting invasion as previously described [46]. This may be related to the so-called ‘go or grow’ hypothesis stating that proliferation and invasion are two mutually exclusive events [46, 47]. The hypothesis states that the cells are unable to commit to cell division and migration simultaneously, hence temporally migration suppress proliferation and vice versa. This indeed seemed to be the case for the GSS cultures T78 and T86 when following the tumor cells over time. The time-lapse movies of invasion suggest that the cells move randomly without any particular direction, however, the cells stall and becomes immobile upon cell division. In the present study this takes place in a stem cell medium-based context thereby confirming a previous observation made using serum containing medium [23].

### Comparison of invasion in vitro and in vivo

Upon comparison of invasion distances and area in vivo and in vitro, T87 had the longest invasion distance and largest invasion area in both models. T78 had the second longest invasion distance in both models closely followed by T86, whereas very limited or no invasion was seen for U87. The mesenchymal subtype has earlier been associated with aggressiveness and increased invasion [48]. Despite this, we identified T87, a proneural culture to be the most invasive. This may be explained by different mechanisms driving invasion within each subtype. To perform a direct comparison of invasion potential associated with subtype more cultures of each subtype should be included in future studies. Only a few studies comparing invasion in vivo and in vitro have been performed previously and to our knowledge not for gliomas. In one study the invasion of throphoblasts in an in vitro three-dimensional co-culture model was performed and found to resemble the in vivo situation [49]. Another study investigated invasion through a matrigel using a series of normal and malignant epithelial and mesenchymal cells. This study showed that matrigel did not provide a universal model for mimicking invasiveness in vivo [50]. Together these studies and the present study seem to suggest that the use of in vivo-like brain matrix like slice cultures for invasion studies are preferable.

### Area and invasion measurements in vitro

We compared spheroid- and invasion area in confocal and immunohistochemical images for the in vitro invasion model. Regarding spheroid area (Figs 3A and 4A) the area in the confocal images appeared a little larger than in the immunohistochemical images. These differences are most likely a result of the confocal images comprising several superimposed images thus corresponding to the total amount of invasive cells, whereas the immunohistochemical images represent a 3 μm section from a certain level in the spheroid. Diffusion of DiI from the spheroid into the brain slice might also be part of the explanation [51]. Such diffusion into the brain tissue could be interpreted as an enlarged area and as invasion. Supporting this, U87 showed invasion in the confocal images (Fig 2A–2C and Fig 3B) but not in the immunohistochemical images (Fig 4A, 4B, 4J and 4K). Thus subsequent immunohistochemical analysis validating the invasion seen in the confocal images is important and studies using only confocal imaging should be interpreted with some caution.

### Phenotypes and models

The stem cell markers chosen in the present study to compare the phenotypes in the three different models were CD133, nestin and podoplanin. When first described only CD133 positive tumor cells were believed to initiate tumors in mice [24]. In the present study the GSS cultures varied in CD133 expression and even though both the T86 and T87 spheroid cultures had almost no CD133 expression they were still capable of tumor initiation in vivo. This is supported by the study by Wang et al. showing tumor initiation by CD133 negative cells [52]. Nestin is expressed in neural stem cells and is believed to be important in proliferation and invasion [53]. Interestingly, nestin was the only stem cell marker expressed by the invasive tumor cells of all three GSS cultures suggesting a function of nestin in these cells, which is in line with previous findings of nestin being important in invasion [53]. Podoplanin was primarily included in the study based on its role in stemness and its potential involvement in invasion [54]. In this study, however, podoplanin was only found in a few invasive cells in the T86 culture.

The tumors in mice seemed to some extend to express more of the stem cell markers than both the cultured spheroids and the spheroids implanted into the brain slice cultures. This may be explained by the complex in vivo microenvironment in the mice brains and mice brain tumors being best at preserving tumor stem cell features [55–57]. The tumor microenvironment is maintained by several factors like VEGF [58], low oxygen levels [59, 60] and niches of tumor stem cells [31, 57, 61] playing an important role in regulation of the stem cells. A functional vascular compartment instead of remnants of vessels being present in the slice cultures [62, 63] may be import for both the VEGF secretion and the function of the perivascular tumor stem cell niche. Another explanation might be the shorter time course of spheroid development and invasion in vitro compared with the longer observation time in vivo. Although these explanations represent potential limitations of the brain slice culture model, expression of the stem cell markers justifies that the model is used in studies focusing on these aspects.

The three GSS cultures were all derived from IDH1-negative tumors but showed differences in their methylation status and karyotypes supporting the different phenotype of the cultures observed in this study. Another important aspect to be stressed is that the GSS cultures most likely are polyclonal leading to growth and propagation of different cell types dependent on the models used. Incorporating thereby the therapeutically relevant heterogeneity of glioblastoma in the established in vitro model, these aspects at the same time suggest that spheroids from different GSS lines should be used for testing novel therapeutic strategies.

### Conclusion and perspectives

In conclusion, the advantage of the in vitro invasion model established in the present study is the in vivo-like invasion and the use of serum-free medium taking the stem-like cell population into account. The model is feasible and robust and as a strong methodological aspect, it is possible to investigate the tumor cell invasion and the cellular expression of various markers using immunohistochemistry. We believe that the established in vivo-like model has a great potential in screening of new anti-cancer drugs including evaluation of anti-invasive effects into the screening.

## Supporting Information

S1 Fig Growth curves for GSS cultures and U87. The GSS cultures and U87 were cultured in serum-free medium as spheroids and trypsinated. Cells were seeded and the cell number estimated in triplicates at day 1–5. The data shown are mean; n = 3. Bars; SEM.
(TIF)

S2 Fig Cell division in time-lapse movie. Still pictures obtained from the time-lapse movie with T86 showing a cell becoming immobile after cell division. The pictures were obtained after 22–24 hours. Arrows show the cell before (A, D), during (B, E) and after cell division (C, F).
(TIF)

S3 Fig Expression of glial and neuronal markers in brain slice cultures. Brain slice cultures were fixed, paraffin embedded, sectioned (3 μm) and immunohistochemically stained for GFAP (A, D), MAP2 (B, E) and NeuN (C, F). Both the cortex and striatum expressed the astrocytic marker GFAP and the neuronal markers MAP2 and NeuN. **Scalebar 100 μm**.
(TIF)

S4 Fig Comparison of Ki-67 expression. Ki-67 expression in immunostained sections of cultured spheroids (A, D, G, J), in vivo xenografts (B, E, H, K) and implanted spheroids (C, F, I, L) from U87 (A-C) and the three GSS cultures (D-L). The outlined areas identify spheroids implanted into the brain tissue (C, F, I, L). Scalebar 100 μm.
(TIF)

S5 Fig Comparison of CD133 expression. CD133 expression in immunostained sections of cultured spheroids (A, D, G, J), in vivo xenografts (B, E, H, K) and implanted spheroids (C, F, I, L) from U87 (A-C) and the three GSS cultures (D-L). The outlined areas identify tumor developed in mice (H) and spheroids implanted into the brain tissue (C, F, I, L). Scalebar 100 μm.
(TIF)

S6 Fig Comparison of nestin expression. Nestin expression in immunostained sections of cultured spheroids (A, D, G, J), in vivo xenografts (B, E, H, K) and implanted spheroids (C, F, I, L) from U87 (A-C) and the three GSS cultures (D-L). The outlined areas identify tumor developed in mice (H) and spheroids implanted into the brain tissue (C, F, I, L). Inserts show area indicated by arrow in higher magnification. Scalebar 100 μm.
(TIF)

S7 Fig Comparison of podoplanin expression. Podoplanin expression in immunostained sections of cultured spheroids (A, D, G, J), in vivo xenografts (B, E, H, K) and implanted spheroids (C, F, I, L) from U87 (A-C) and the three GSS cultures (D-L). The outlined areas identify tumor developed in mice (H) and spheroids implanted into the brain tissue (C, F, L). Inserts show area indicated by arrow in higher magnification. Scalebar 100 μm.
(TIF)
