# Supplementary figures and images for: Establishment and Characterization of a Tumor Stem Cell-Based Glioblastoma Invasion Model
Source: PLoS One. 2016 Jul 25;11(7):e0159746. doi: 10.1371/journal.pone.0159746 (PMC4959755; doi:10.1371/journal.pone.0159746)

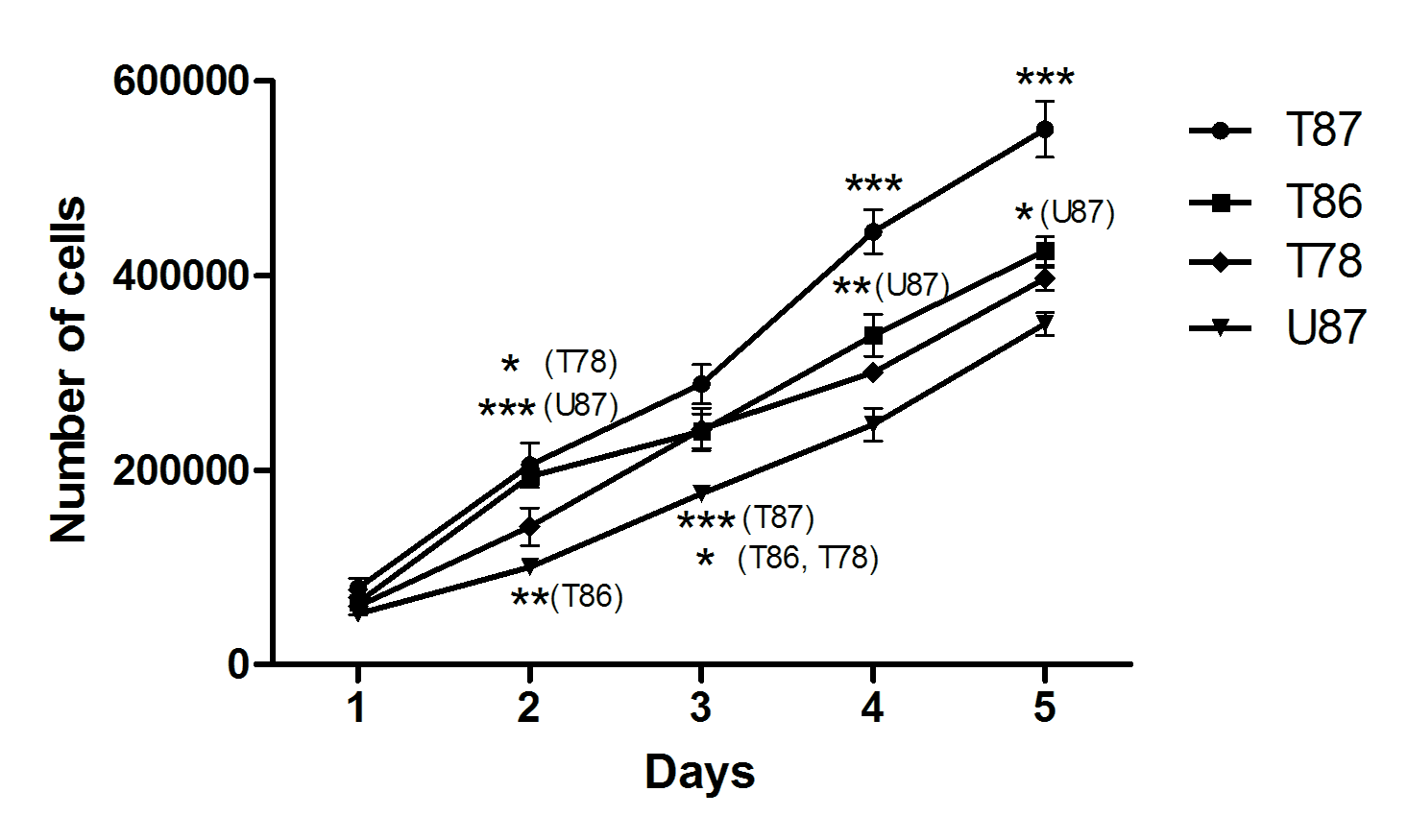

Supplement: S1 Fig — The GSS cultures and U87 were cultured in serum-free medium as spheroids and trypsinated. Cells were seeded and the cell number estimated in triplicates at day 1–5. The data shown are mean; n = 3. Bars; SEM. (TIF) [file pone.0159746.s001.tif]

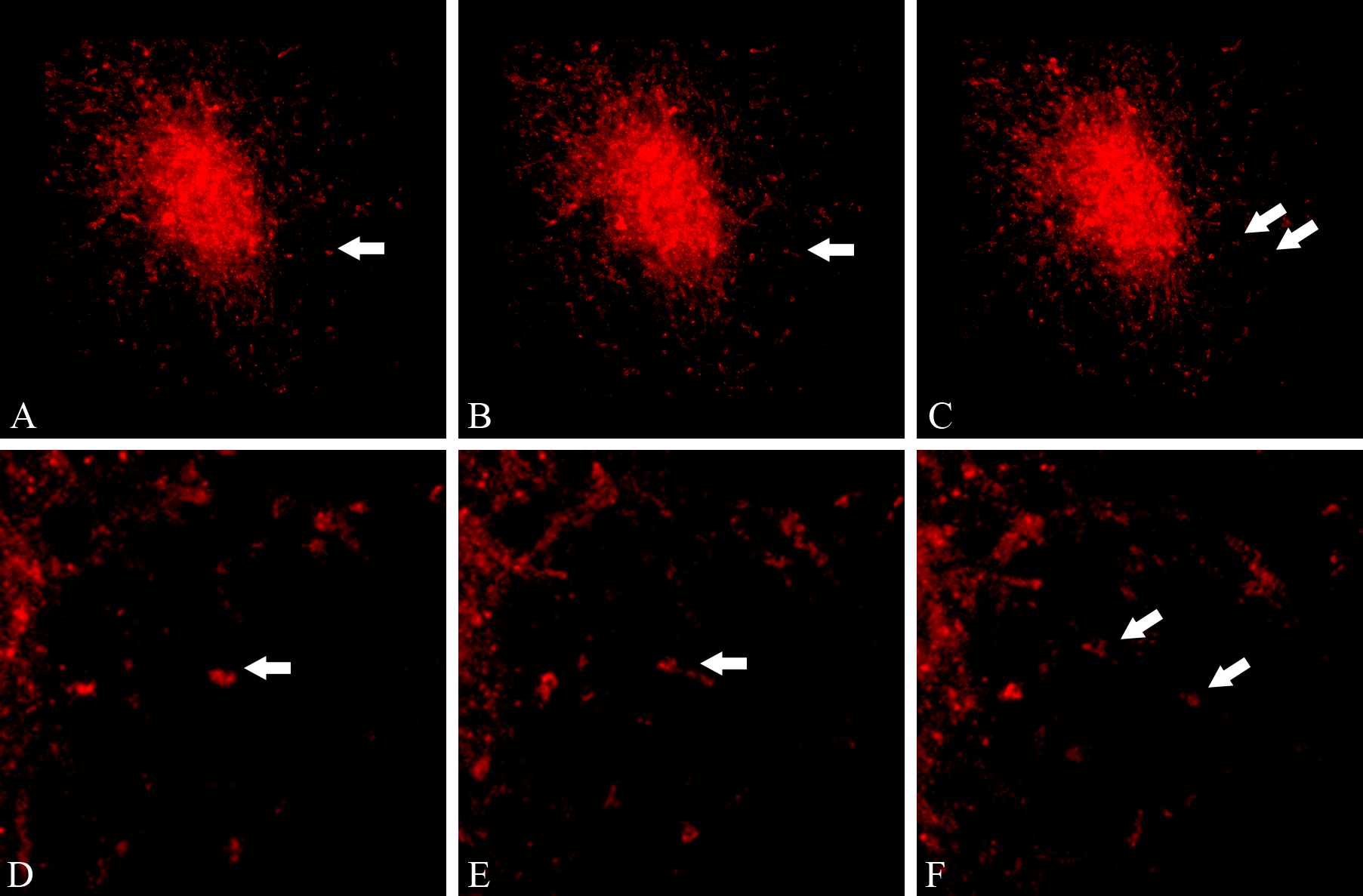

Supplement: S2 Fig — Still pictures obtained from the time-lapse movie with T86 showing a cell becoming immobile after cell division. The pictures were obtained after 22–24 hours. Arrows show the cell before (A, D), during (B, E) and after cell division (C, F). (TIF) [file pone.0159746.s002.tif]

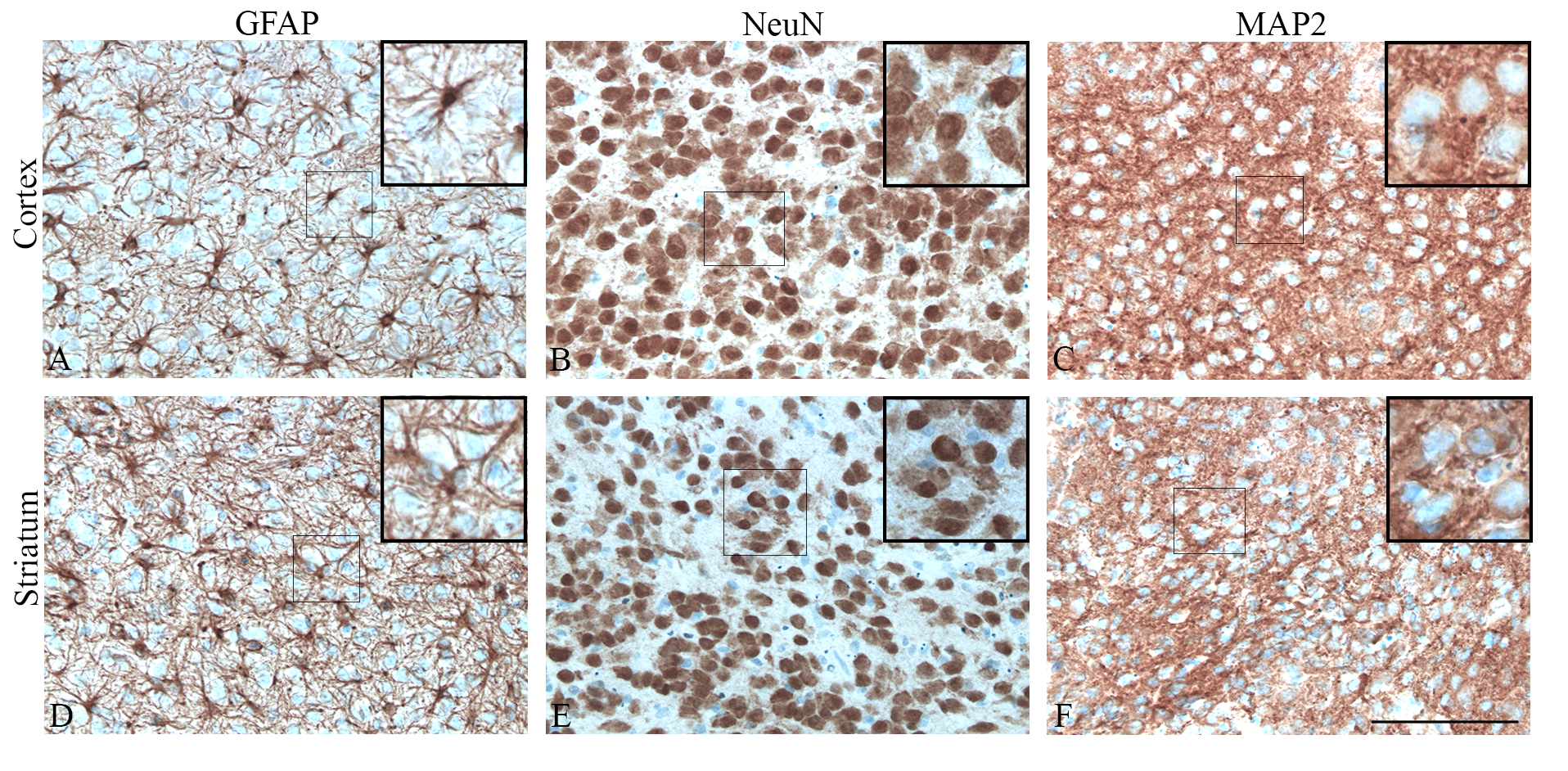

Supplement: S3 Fig — Brain slice cultures were fixed, paraffin embedded, sectioned (3 μm) and immunohistochemically stained for GFAP (A, D), MAP2 (B, E) and NeuN (C, F). Both the cortex and striatum expressed the astrocytic marker GFAP and the neuronal markers MAP2 and NeuN. Scalebar 100 μm. (TIF) [file pone.0159746.s003.tif]

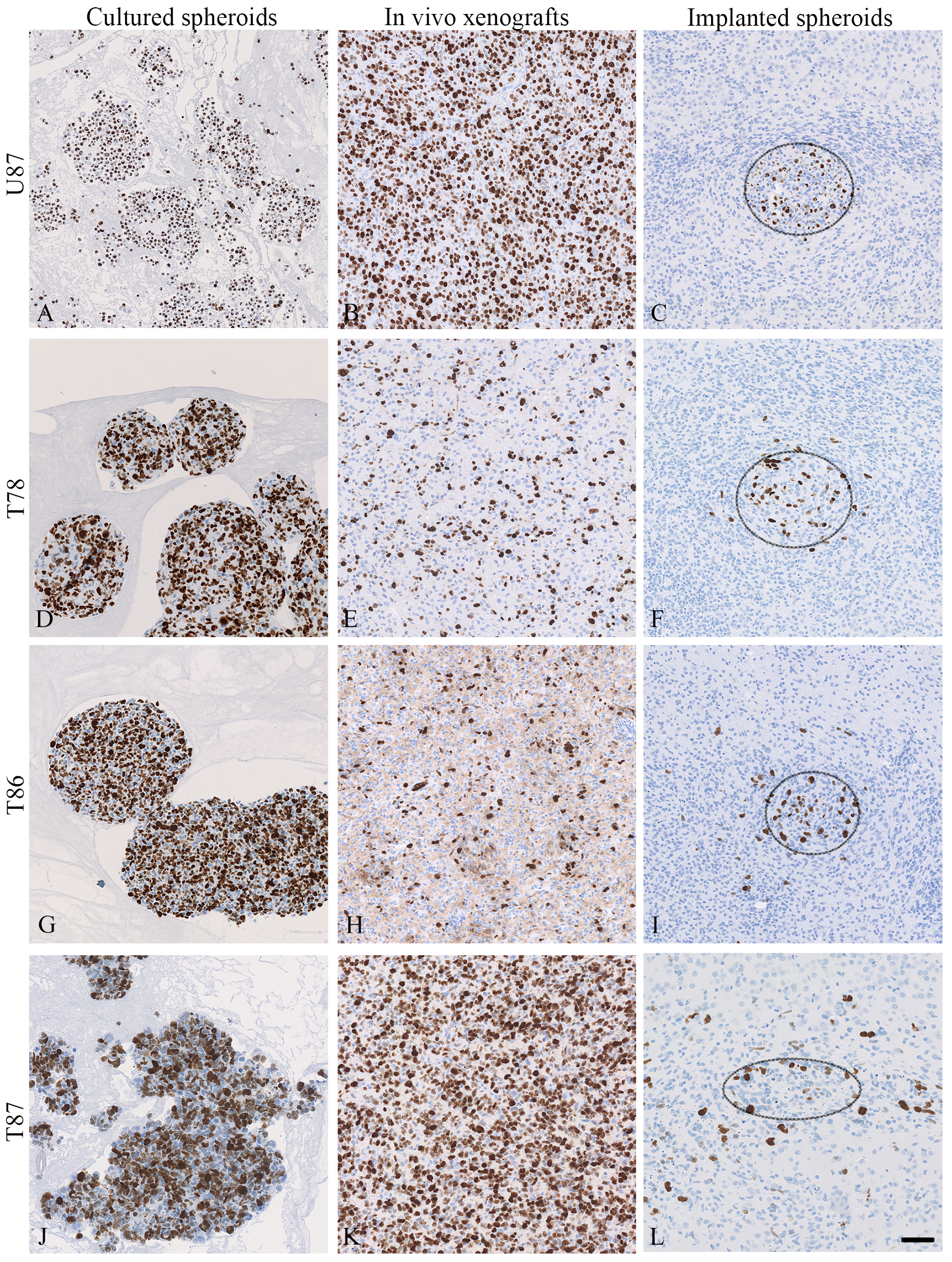

Supplement: S4 Fig — Ki-67 expression in immunostained sections of cultured spheroids (A, D, G, J), in vivo xenografts (B, E, H, K) and implanted spheroids (C, F, I, L) from U87 (A-C) and the three GSS cultures (D-L). The outlined areas identify spheroids implanted into the brain tissue (C, F, I, L). Scalebar 100 μm. (TIF) [file pone.0159746.s004.tif]

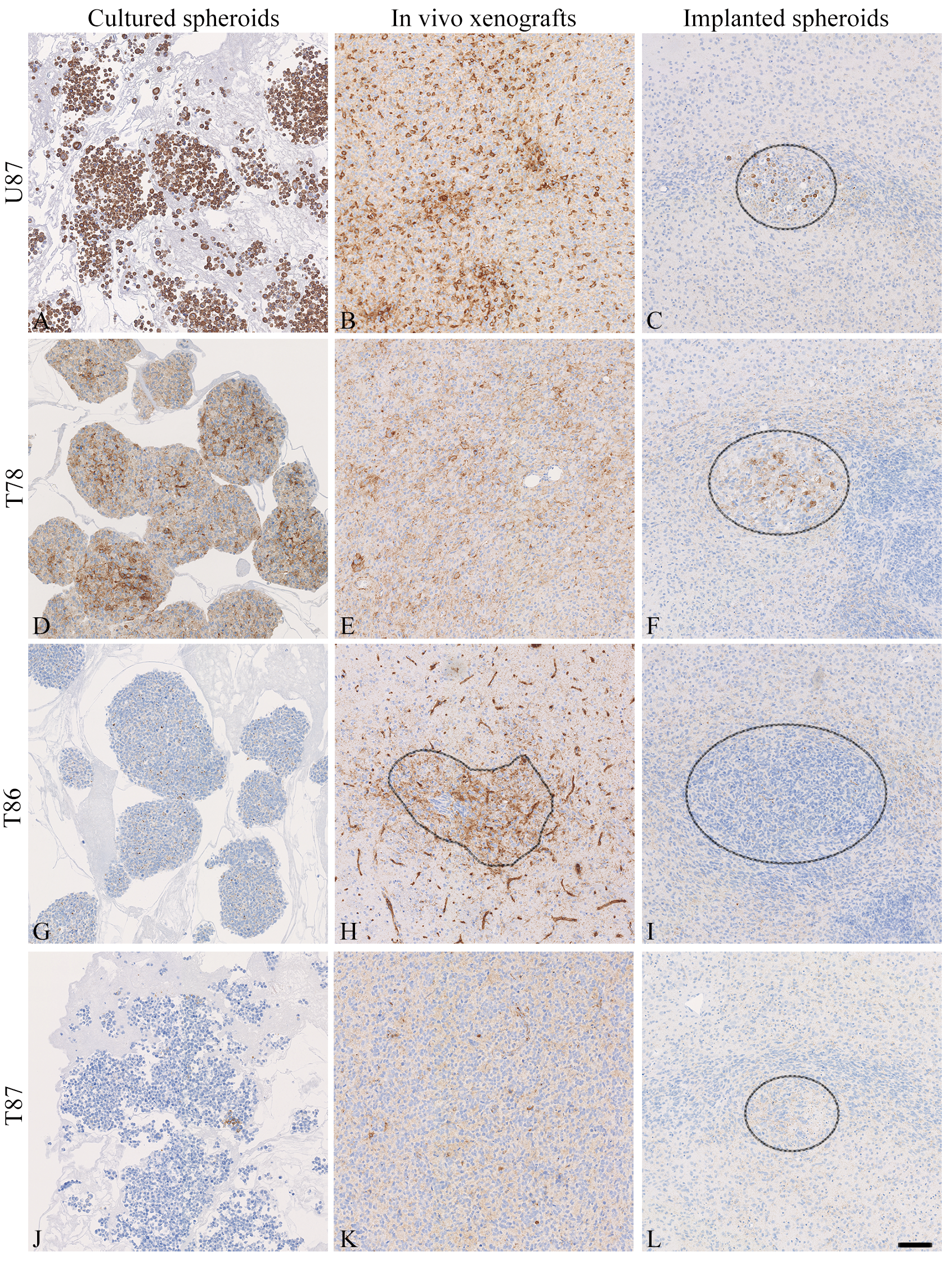

Supplement: S5 Fig — CD133 expression in immunostained sections of cultured spheroids (A, D, G, J), in vivo xenografts (B, E, H, K) and implanted spheroids (C, F, I, L) from U87 (A-C) and the three GSS cultures (D-L). The outlined areas identify tumor developed in mice (H) and spheroids implanted into the brain tissue (C, F, I, L). Scalebar 100 μm. (TIF) [file pone.0159746.s005.tif]

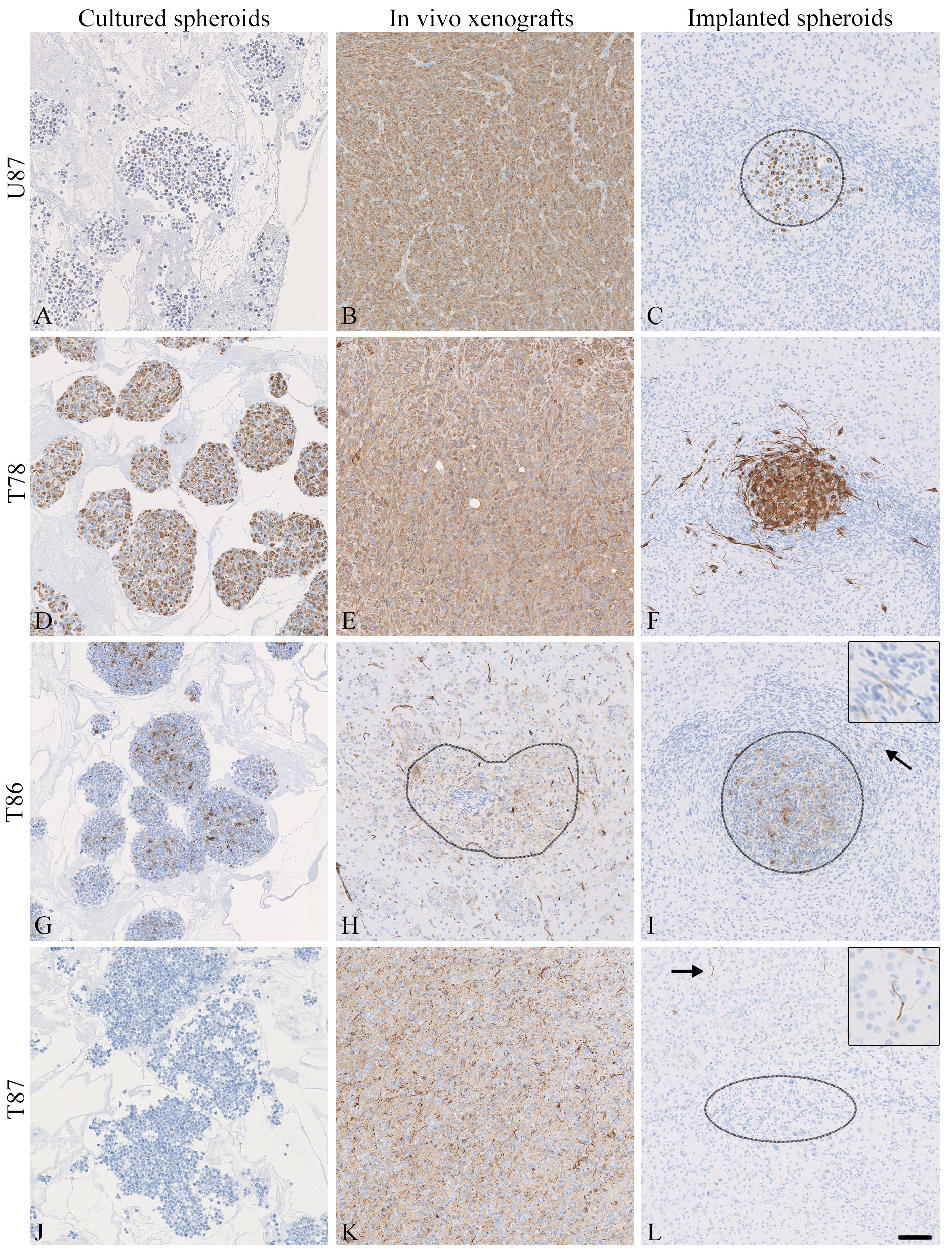

Supplement: S6 Fig — Nestin expression in immunostained sections of cultured spheroids (A, D, G, J), in vivo xenografts (B, E, H, K) and implanted spheroids (C, F, I, L) from U87 (A-C) and the three GSS cultures (D-L). The outlined areas identify tumor developed in mice (H) and spheroids implanted into the brain tissue (C, F, I, L). Inserts show area indicated by arrow in higher magnification. Scalebar 100 μm. (TIF) [file pone.0159746.s006.tif]

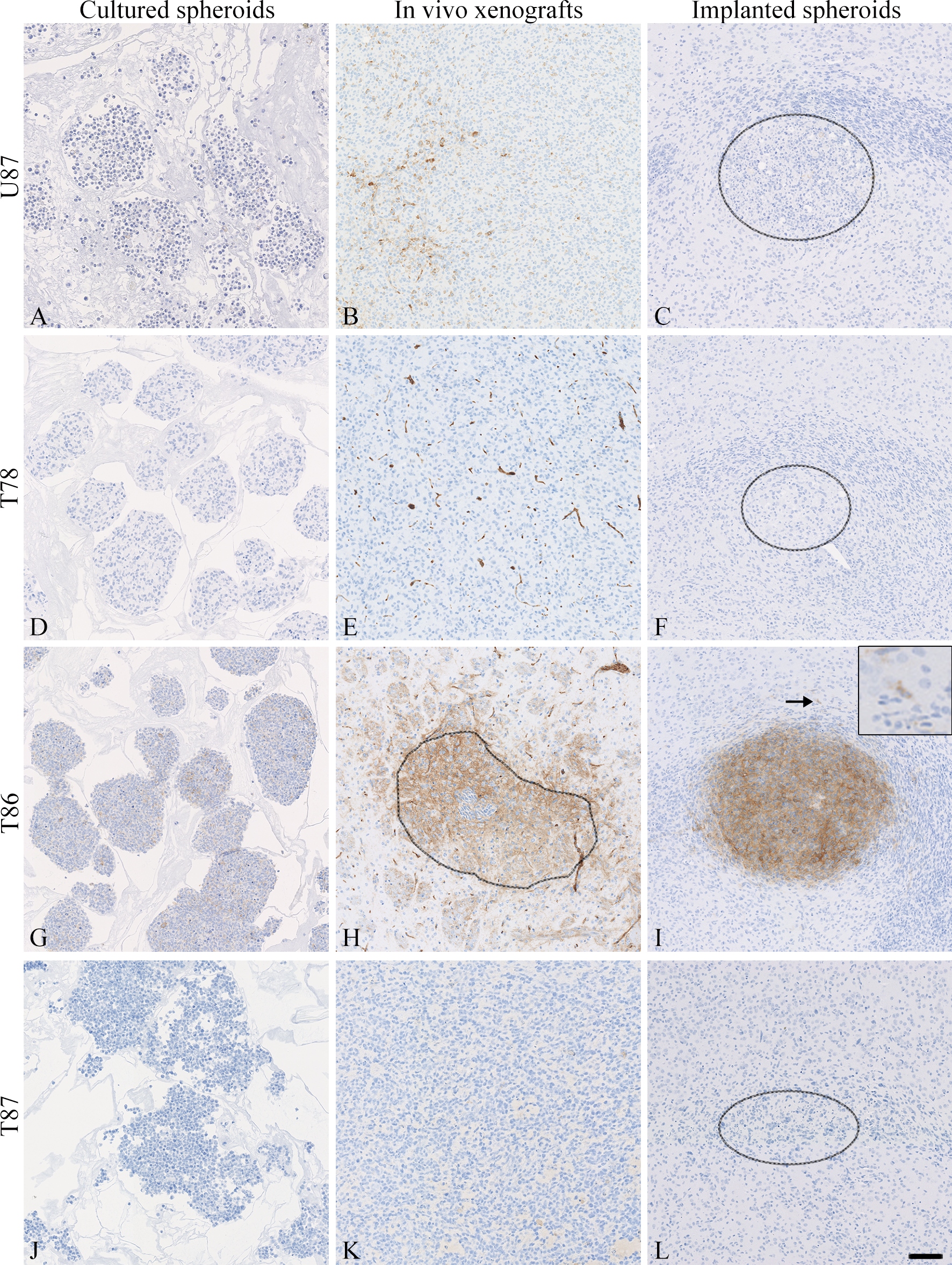

Supplement: S7 Fig — Podoplanin expression in immunostained sections of cultured spheroids (A, D, G, J), in vivo xenografts (B, E, H, K) and implanted spheroids (C, F, I, L) from U87 (A-C) and the three GSS cultures (D-L). The outlined areas identify tumor developed in mice (H) and spheroids implanted into the brain tissue (C, F, L). Inserts show area indicated by arrow in higher magnification. Scalebar 100 μm. (TIF) [file pone.0159746.s007.tif]
